# Comparison of linear and non-linear machine learning models for time-dependent readmission or mortality prediction among hospitalized heart failure patients

**DOI:** 10.1016/j.heliyon.2023.e16068

**Published:** 2023-05-06

**Authors:** Rui Tong, Zhongsheng Zhu, Jia Ling

**Affiliations:** Department of Cardiology, Shanghai Pudong Hospital, Fudan University Pudong Medical Center, Shanghai, 201399, China

**Keywords:** Heart failure, Readmission and mortality, Survival analysis, Machine learning

## Abstract

Although many models are available to predict prognosis of heart failure patients, most tools combining survival analysis are based on proportional hazard model. Non-linear machine learning algorithms would overcome the limitation of the time-independent hazard ratio assumption and provide more information in readmission or mortality prediction among heart failure patients.

The present study collected the clinical information of 1796 hospitalized heart failure patients surviving during hospitalization in a Chinese clinical center from December 2016 to June 2019. A traditional multivariate Cox regression model and three machine learning survival models were developed in derivation cohort. Uno's concordance index and integrated Brier score in validation cohort were calculated to evaluate the discrimination and calibration of different models. Time-dependent AUC and Brier score curves were plotted to assess the performance of models at different time phases.

## 1 Introduction

Heart failure is a frequent cause of hospitalization and place a heavy burden on the individuals, their families and the society [1]. Reducing the high rate of readmission among discharged heart failure patients would improve their outcomes and decrease costs [2]. Thus, it is critically important for patients and health care providers to have access to an accurate and reliable prognosis prediction tool. Nevertheless, developing a readmission or mortality prediction model among hospitalized heart failure patients suitable for hospital settings at different quality supervision levels has proven to be a difficult task. Although many efforts have been made to establish available tools such as ADHERE [3], LaCE [4], RR [[5], [6], [7]] and Redin Score [8], most of them are based on models for endpoints at particular isolated time points. Only a few prognosis prediction models derived from cohorts in developed and high income countries took survival data and censored data into account. These studies, respectively conducted in Australia, Germany and Netherland, reported concordance indices ranging from 0.63 to 0.80 as the assessment of multivariate Cox regression models based on the assumption of constant hazard ratio of variables over time [[9], [10], [11]]. However, the high-dimensional and non-linear relationship between clinical data and outcomes could not be identified effectively in the framework of conventional statistic methods.

Machine learning, a widely accepted computational technique in processing high-dimensional data and non-linear relationship, may help to capture the subtle relevance among variables and outcomes and achieve better model performance than conventional statistic methods [12,13]. Many supervised learning methods not based on neural networks, sometimes called ‘traditional machine learning’, including decision tree, support vector machine, Bayesian learning, naïve Bayes, K nearest neighbor algorithm and gradient boosting [14,15], are applied to prediction tasks such as forecasting and classification [16]. Compared with the powerful but time-consuming and computational expensive deep learning, these traditional machine learning methods are much faster to develop and test on a given problem, while requiring less amounts of data [17]. As statisticians and mathematicians have explored the ranked set sampling method to estimate variables subject to exponential distribution [18,19], it seems to be possible to combine machine learning algorithms with survival analysis. We conducted a retrospective study based on a clinical dataset in China to derive readmission or mortality prediction models via conventional statistics and machine learning algorithms combined with survival analysis, aiming to ameliorate our knowledge deficiency in time-dependent heart failure prognosis prediction, especially in the case of middle-income countries.

## 2 Materials and methods

### 2.1 Study participants

**Fig. 1:**
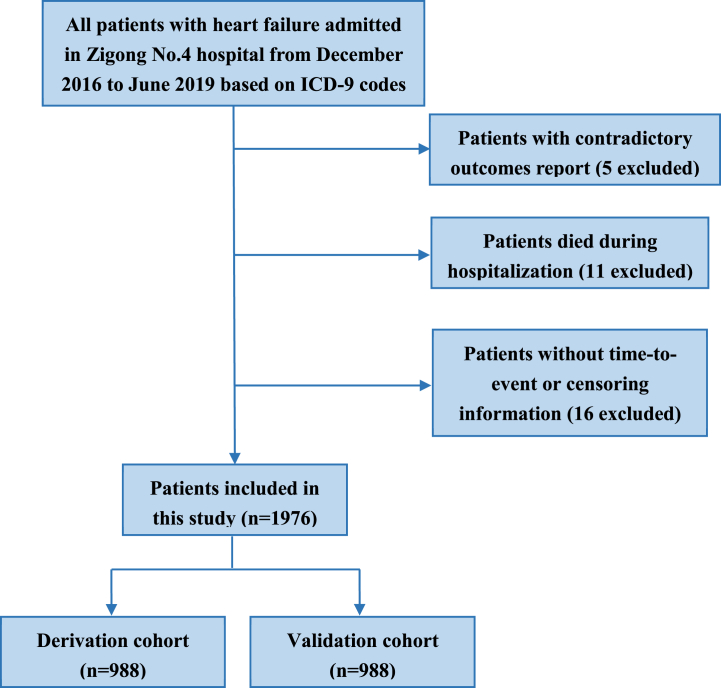
The flow chart for patient inclusion and cohort splitting.

The data for the derivation and validation of survival models were downloaded from PhysioNet (https://doi.org/10.13026/8a9e-w734) (version 1.2) on December 5th, 2021, originating from a retrospective study based on electronic healthcare records (EHR) conducted at Zigong Fourth People's Hospital [20]. The study was approved by the ethics committee of Zigong Fourth People's Hospital (Approval Number:2020-010). From December 2016 to June 2019, all discharged patients diagnosed with heart failure on hospital admission at Zigong Fourth People's Hospital were enrolled in our analysis. The diagnosis of heart failure was identified by ICD-9 codes 428.23, 428.3, 428.30, 428.31, 428.32, 428.33, 428.4, 428.40, 428.41, 428.42, 428.43, 428.9 in the EHR. Electronic medical records of these enrolled patients were reviewed to confirm that they fulfilled the definition of heart failure according to the European Society of Cardiology (ESC) criteria [21]. Participants were excluded from our analysis if they had contradictory outcome report according to EHR and follow-up data, or they died during hospitalization. Patients without time-to-event or censoring information were also excluded. The flowchart of participant inclusion and exclusion is showed in Fig. 1.

### 2.2 Outcome definition

Readmission or mortality was defined as the primary composite end point in our analysis. Mortality was defined as death due to any cause, and readmission was defined as unplanned hospital readmission due to advanced decompensated heart failure. Survival time, namely time to death or readmission, defined as days from index hospital admission to the primary end point. Patients who didn't suffer the primary end point during the follow-up were labels as censored data.

### 2.3 Candidate variables and data preprocessing

The origin variables extracted from EHR included several categories. Demographic data were collected by the nurses on admission, while baseline clinical characteristics were measured on the day of hospital admission. Comorbidities in medical history included the items in Charlson Comorbidity Index (CCI), and laboratory findings were obtained from day of hospital admission. Medications administered during hospitalization were also recorded as candidate predict variables for prognosis outcomes. Binary variables and polytomous ranked variables were converted simply by an ordinal encoder in scikit-learn, while polytomous nominal variables were transformed into dummy variables by a one hot encoder. Duplicated variables and redundant variables with perfect collinearity based on clinical knowledge were reviewed and eliminated after an agreement was reached among all authors.

Outliers identified according to clinical knowledge were labeled as missing values. Variables with missing value rates higher than 70% were excluded in model derivation and validation. For variables with missing value rates lower than 30%, missing values were interpolated by SimpleImputer with basic strategies such as mean imputation for continuous variables in line with normal distribution, median imputation for continuous variables with skew distribution and mode imputation for categorical variables. As to variables with missing value rates ranging from 30% to 70%, the two authors both of whom are cardiologists reviewed the published literatures of previous prediction models and assessed the clinical importance of these variables. The decision of whether a specific variable should be excluded or retained for further analysis was made after a consultation between the two authors.

### 2.4 Model derivation and validation

The dataset was randomly divided into two cohorts containing equal numbers of participants: one for prediction model derivation, and the other cohort for internal validation. With the variables showing statistically significant relevance (p < 0.05) to prognosis outcomes in univariate Cox regression, a conventional statistic method based on the assumption of constant hazard ratio over time, known as multivariate Cox regression, was established as a reference baseline model. A penalized Cox model based on L1 regularization [22] and two non-linear survival machine learning models respectively based on random survival forest [23,24] and gradient boosting survival analysis [25] were developed in derivation cohort. The grid search strategy based on a five-fold cross validation framework in derivation cohort was adopted for selection optimized hyperparameters of machine learning models, aiming to achieve the highest mean concordance index. The adjusted hyperparameters in model optimization include max depth, min samples split and min samples leaf in random survival forest model; learning rate, number of estimator and subsample rate in gradient boosting survival models; and the penalty strength α in Least Absolute Shrinkage and Selection Operator (LASSO) Cox model. The permutation importance [26] of every selected variable of two tree-based models, defined as the mean reduction of model performance when the observations of particular variable in derivation cohort were randomly permutated in a series of repeat tests, was calculated as a measurement of the factor's contribution to prognosis prediction among heart failure patient. For each tree-based algorithms, different importance thresholds for variables inclusion were tried until the subset of variables achieved a highest c-index in derivation cohort. The selected best subsets based on feature importance and best hyperparameter combinations constituted the final models and their practical performance was tested in the validation cohort.

### 2.5 Model performance evaluation

The fixed models were evaluated in discrimination and calibration aspects. For model discrimination comparison, Uno's concordance index, rather than Harrell's concordance index, was calculated due to the moderate amounts of censoring [27]. As an extension of the receiver operating characteristics (ROC) curve to continuous outcomes such as survival time, the time-dependent cumulative/dynamic ROC [28] based on cumulative cases who experienced an event prior to or at time *t* (*t*_*i*_ ≤ *t*) and dynamic controls with *t*_*i*_ > *t* was used to assess the prediction performance of different models in the occurrence of the endpoint in a period up to time *t*. The areas under ROC curves (AUC) at 30 different time points during follow-up were calculated and time-dependent AUC curves were plotted for visualization of model discrimination in different time intervals. The time-dependent Brier Score (${BS}^{c}\left(t\right)$), as an extension of the mean squared error to right censored data, was calculated for model calibration assessment. It is defined as:

$${BS}^{c}\left(t\right)=\frac{1}{n}\sum_{i=1}^{n}I\left(y_{i}\leq t⋀\delta_{i}=1\right)\frac{{\left(0-\hat{\pi }\left(t|x_{i}\right)\right)}^{2}}{\hat{G}\left(y_{i}\right)}+I\left(y_{i}>t\right)\frac{{\left(1-\hat{\pi }\left(t|x_{i}\right)\right)}^{2}}{\hat{G}\left(t\right)}$$

where $\hat{\pi }\left(t|x\right)$ is a model's predicted probability of remaining event-free up to time point *t* for feature vector *x*, and $1/\hat{G}\left(t\right)$ is an inverse probability of censoring weight. The integrated Brier score (IBS) defined as

$$IBS=\int_{t_{1}}^{t_{\max}}{BS}^{c}\left(t\right)dw\left(t\right)$$

where the weighting function is $w\left(t\right)=t/t_{\max}$, was also estimated via the trapezoidal rule for overall evaluation of model calibration at all available times $t_{1}\leq t\leq t_{\max}$ [29].

### 2.6 Statistics

Kolmogorov-Smirnov test was conducted to assess the normality of variables. Continuous variables with normal distributions were presented as mean ± standard deviation (SD), while continuous variables with skew distribution were reported with mean and a range from the 1st quantile to the 3rd quantile (Q1-Q3). Categorical variables were presented as proportions. Student's t tests for continuous variables were performed between two normally distributed groups with homogeneity of variance. Wilcoxon rank sum tests were used for the identification of statistic differences of continuous variables between two groups with skew distribution, as well as polytomous ranked categorical variables. The differences of nominal variables between two groups were evaluated by Chi-square test. The 95% confident intervals (95% CI) of Uno's concordance indices and integrated Brier scores in validation cohort were obtained via bootstrap resampling method.

All analyses were completed in Jupyterlab 2.2.6 under the environment of Anaconda3 (version 2020.11) based on the code of Python (version 3.8.5). The statistic module was from a package of scipy (version 1.5.2) and the machine learning module was from packages in scikit-learn (version 0.23.2). Survival analysis was performed with a combination of the package scikit-survival (version 0.17.2) and lifelines (version 0.27.0). Tests were two-sided and p values < 0.05 were considered statistically significant.

## 3 Results

### 3.1 Participants characteristics

**Fig. 2:**
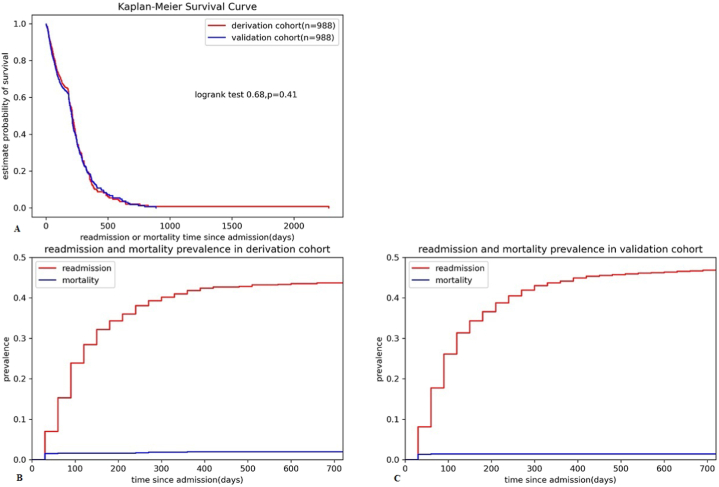
The Kaplan-Meier survival curves and readmission or mortality prevalence curves in derivation and validation cohorts. (A) The Kaplan-Meier survival curve in derivation and validation cohort. (B) Readmission or mortality prevalence in derivation cohort. (C) Readmission or mortality prevalence in validation cohort.

1976 hospitalized patients with heart failure were enrolled in our analysis after a review of all 2008 participants in the original dataset. These participants were randomly divided into two groups: 988 patients constituted a cohort for model derivation and the rest formed a validation cohort. The baseline characteristics of patients are listed in Table 1. No significant differences between derivation and validation cohort on demographic characteristics of gender, age, occupation, discharge destination and clinical severity features such as admission way, admission ward, NYHA classification, Killip grade, type of heart failure, LVEF and serum creatinine levels. During the follow-up, 451 patients (45.6%) in derivation cohort and 480 patients (48.6%) in validation cohort suffered the primary end point, and there was no difference of outcome incidence and right censoring proportions between two cohorts (χ^2^ = 1.59, p = 0.21). Kaplan-Meier survival curves of derivation and validation cohort were plotted in Fig. 2, showing statistic equivalent survival time estimation between two cohorts (Log-rank statistic = 0.68, p = 0.41). The separate accumulated prevalence curves of readmission and mortality in derivation and validation cohort were also presented in Fig. 2.

**Table 1:** Baseline level of demographic and clinical variables in derivation and validation cohort.

Variables	Total (n = 1976)	Derivation (n = 988)	Validation (n = 988)	P value
Gender				
Male (n, %)	827 (41.9)	418 (42.3)	409 (41.4)	0.72
Female (n, %)	1149 (58.1)	570 (57.7)	579 (58.6)	
Age, median (Q1-Q3),years	74.5 (64.5–84.5)	74.5 (64.5–84.5)	74.5 (64.5–84.5)	0.33
Admission way				
Emergency (n, %)	934 (47.3)	474 (48.0)	460 (46.6)	0.56
Nonemergency (n, %)	1042 (52.7)	514 (52.0)	528 (53.4)	
Admission ward				
Cardiology (n, %)	1521 (77.0)	776 (78.5)	745 (75.4)	0.30
ICU (n, %)	14 (0.7)	8 (0.8)	6 (0.6)	
Generalward (n, %)	261 (13.2)	118 (11.9)	143 (14.5)	
Others (n, %)	180 (9.1)	86 (8.7)	94 (9.5)	
Discharge destination				
Home (n, %)	1333 (67.5)	691 (69.9)	642 (65.0)	0.06
Healthcare facility (n, %)	435 (22.0)	199 (20.1)	236 (23.9)	
Others (n, %)	208 (10.5)	98 (9.9)	110 (11.1)	
Occupation				
Urban resident (n, %)	1645 (83.2)	810 (82.0)	835 (84.5)	0.39
Farmer (n, %)	194 (9.8)	105 (10.6)	89 (9.0)	
Officer (n, %)	7 (0.4)	2 (0.2)	5 (0.5)	
Worker (n, %)	17 (0.9)	9 (0.9)	8 (0.8)	
Others (n, %)	113 (5.7)	62 (6.3)	51 (5.2)	
NYHA classification				
Grade II (n, %)	346 (17.5)	183 (18.5)	163 (16.5)	0.37
Grade III (n, %)	1025 (51.9)	508 (51.4)	517 (52.3)	
Grade IV (n, %)	605 (30.6)	297 (30.1)	308 (31.2)	
Killip grade				
Grade I (n, %)	524 (26.5)	248 (25.1)	276 (27.9)	0.70
Grade II (n, %)	1017 (51.5)	531 (53.7)	486 (49.2)	
Grade III (n, %)	383 (19.4)	183 (18.5)	200 (20.2)	
Grade IV (n, %)	52 (2.6)	26 (2.6)	26 (2.6)	
Myocardial infarction (n, %)	140 (7.1)	69 (7.0)	71 (7.2)	0.93
Type of heart failure				
Left (n, %)	471 (23.8)	239 (24.2)	232 (23.5)	0.71
Right (n, %)	51 (2.6)	28 (2.8)	23 (2.3)	
Both (n, %)	1454 (73.6)	721 (73.0)	733 (74.2)	
LVEF, median (Q1-Q3), %	51 (41–61)	50 (41–61)	52 (41.5–61.5)	0.36
Creatinine, median (Q1-Q3), μmol/L	86.8 (65.0–121.5)	87.2 (64.7–121.6)	86.0 (65.2–121.4)	0.52

### 3.2 Variables for prediction

**Fig. 3:**
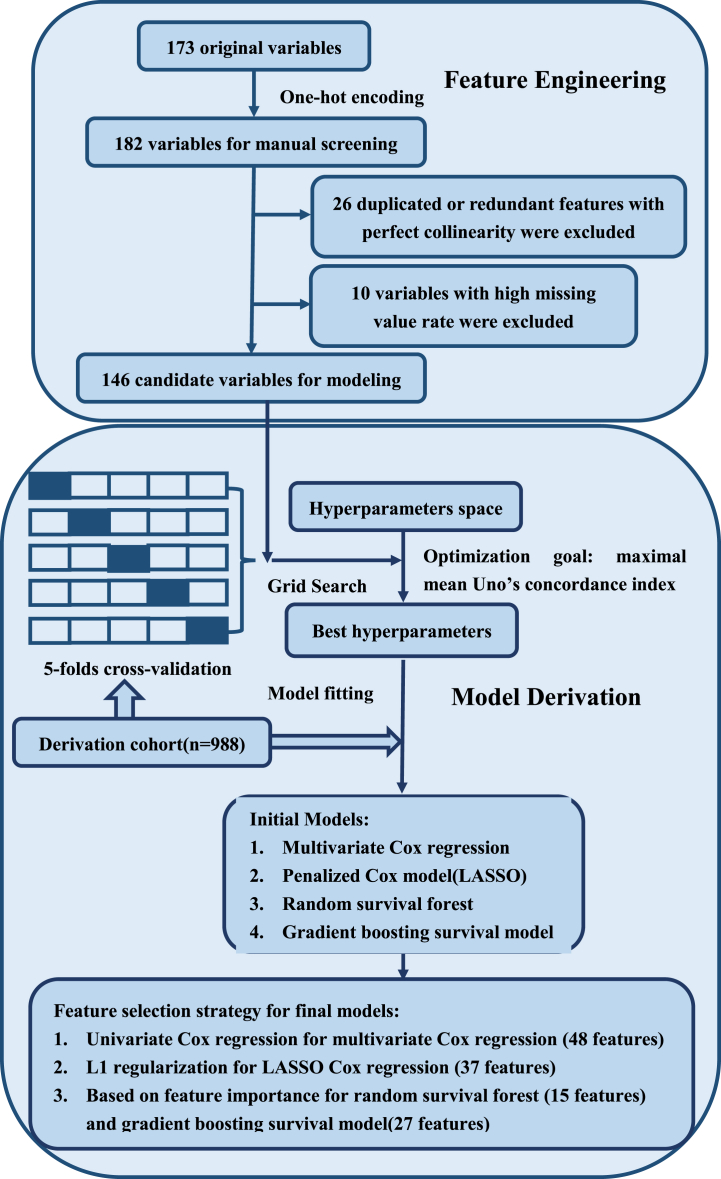
The flow chart for feature engineering and model derivation.

As shown in the flow chart in Fig. 3, there were 173 items as candidate variables in the original dataset. The list of candidate variables was expanded to 182 items after one-hot encoding. Among the candidate variables, 26 items were identified as duplicated features or redundant features having perfect collinearity with other variables based on clinical knowledge, thus excluded from further analysis. After a filtration of features with high missing value rate, 146 predictor variables were retained for further feature selection. The specific missing conditions of each variable and the imputation method used are listed in Supplementary tab. 1. Through univariate Cox regression, 48 variables were identified as predictors in a traditional regression framework, including 4 demographic features, 10 basic clinical features on admission, 3 comorbidities, 5 medications administrated during hospitalization and 26 laboratory or echocardiography findings. The hazard ratios of these selected variable in univariate Cox regression and their adjusted hazard ratios in multivariate Cox regression were listed in Supplementary tab. 2. As a result of dimensional reduction via LASSO regularization, 37 variables were retained for LASSO Cox regression model establishment. After the enumeration of different permutation importance thresholds for variable inclusion, the top 15 most important variables were selected for random survival forest modeling and the top 27 most important features were included for gradient boosting survival analysis as this scale of prediction variables set achieved the highest c-index respectively in derivation cohort.

### 3.3 Model discrimination

**Fig. 4:**
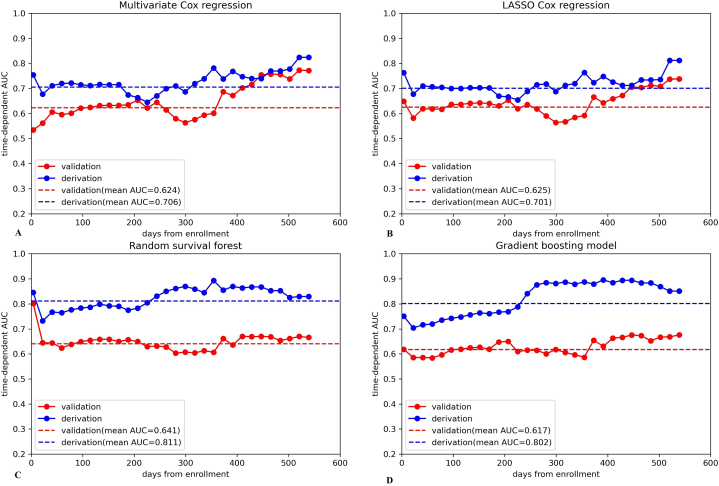
Time-dependent AUC curves of derivation and validation cohorts in different survival prediction models.(A) Time-dependent AUC curves in multivariate Cox regression. (B) Time-dependent AUC curves in LASSO Cox regression. (C) Time-dependent AUC curves in random survival forest. (D) Time-dependent AUC curves in gradient boosting model.

The theoretically discrimination of three machine learning algorithms was compared with the mean Uno's concordance index of the five-fold cross-validation in derivation cohort, which was also considered as the optimization target of model hyperparameters. In LASSO Cox regression, a regularization penalty strength α = 0.001789 reduced the size of predictors to 37 variables and achieved a highest mean Uno's concordance index of 0.605. For random survival forest algorithm, the best mean Uno's c-index was 0.587 with a combination of hyperparameters that “max_depth = 5, min_samples_leaf = 3 and min_samples_split = 2”, while for gradient boosting model, the highest mean Uno's c-index of 0.611 was achieved when the hyperparameters were set as “learning_rate = 0.5, n_estimators = 10 and sub_sample = 0.6”. The practical discrimination of the three machine learning algorithms and the conventional multivariable Cox regression in derivation and validation cohort were listed in Table 2. For scanning the prediction performance in particular time points or intervals, the time-dependent AUC curves of different models in derivation and validation cohort were plotted in Fig. 4.

**Table 2:** Discrimination of different models in derivation and validation cohort.

Models	Uno's concordance index		
	Mean in cross-validation	Derivation (95%CI)	Validation (95%CI)
Multivariate Cox regression	–	0.654 (0.613–0.690)	0.589 (0.557–0.621)
LASSO Cox regression	0.605	0.648 (0.609–0.682)	0.594 (0.563–0.627)
Random survival forest	0.587	0.731 (0.709–0.758)	0.589 (0.556–0.620)
Gradient boosting model	0.611	0.732 (0.706–0.769)	0.582 (0.559–0.607)

### 3.4 Model calibration

**Fig. 5:**
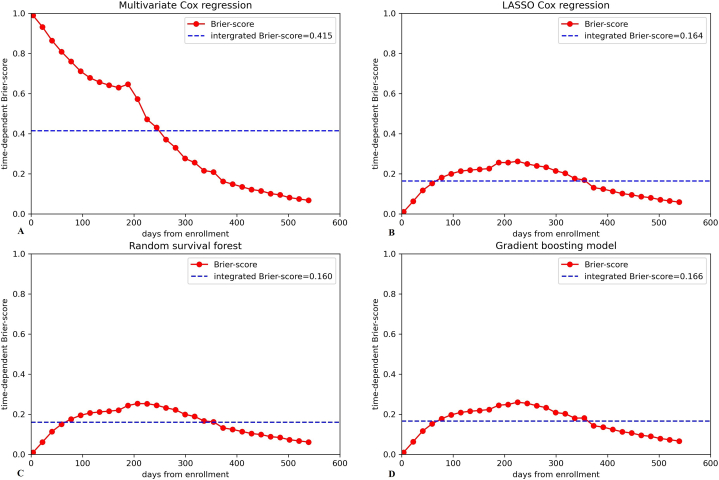
Time-dependent Brier score in different models. (A) Time-dependent Brier score in multivariate Cox regression. (B) Time-dependent Brier score in LASSO Cox regression. (C) Time-dependent Brier score in random survival forest. (D) Time-dependent Brier score in gradient boosting model.

As a reference baseline, a random prediction model without any statistical learning displayed an IBS of 0.264 (95% CI 0.240–0.288) in validation cohort. Another reference baseline model based on Kaplan-Meier estimator that does not consider any variables presented an IBS of 0.167 (95% CI 0.145–0.190). The conventional Cox regression model showed worse calibration ability in validation cohort than baselines, with an IBS of 0.415 (95% CI 0.372–0.465). The IBS (95%CI) of LASSO Cox regression, random survival forest and gradient boosting model were 0.164(0.145–0.188), 0.160(0.138–0.183) and 0.166 (0.140–0.194) respectively. The time-dependent Brier score curves of different models in validation cohort were plotted in Fig. 5.

### 3.5 Importance of factors for prediction in different models

After a procedure of 15 permutation iterations for each selected variable, the top 10 factors with highest permutation importance in multivariate regression and LASSO Cox regression were listed in Table 3. The acid-base equilibrium index in blood gas analysis played a key role in traditional multivariate regression model, while creatinine and systolic blood pressure were the most critical contributors in LASSO Cox model. The top 10 most important predictors in two tree-based models were listed in Table 4. The left ventricular end diastolic diameter (LVEDD) measured by echocardiography, red blood count in routine blood test, uric acid and cholesterol displayed excellent predictive value in both the random survival forest and gradient boosting models.

**Table 3:** The permutation importance of top 10 contribution factors in regression models.

Rank	Multivariate Cox Regression		LASSO Cox Regression	
	Predictors	Importance	Predictors	Importance
1	Measured bicarbonate	0.1741	Creatinine	0.0190
2	Measured residual base	0.1531	Systolic blood pressure	0.0149
3	Standard residual base	0.1355	Right heart failure	0.0134
4	pH	0.1178	Age	0.0108
5	Standard bicarbonate	0.0967	Occupation: farmer	0.0107
6	Total carbon dioxide	0.0472	Congestive heart failure	0.0100
7	Creatinine	0.0193	LVEDD	0.0089
8	Systolic blood pressure	0.0133	Standard deviation of RDW	0.0087
9	Right heart failure	0.0131	Carboxyhemoglobin	0.0076
10	Red blood cell	0.0119	Valsartan tablet	0.0075

LVEDD: left ventricular end diastolic diameter; RDW red blood cell distribution width.

**Table 4:** The permutation importance of top 10 contribution factors in tree-based models.

Rank	Random Survival Forest		Gradient Boosting Survival Model	
	Predictors	Importance	Predictors	Importance
1	LVEDD	0.0410	LVEDD	0.0324
2	Red blood cell	0.0293	Creatinine	0.0232
3	Uric acid	0.0211	Cholesterol	0.0189
4	Urea	0.0202	Red blood cell	0.0174
5	Mean platelet volume	0.0188	Systolic.blood.pressure	0.0127
6	Cholesterol	0.0131	Uric acid	0.0119
7	Right heart failure	0.0128	Mean platelet volume	0.0100
8	DischargeDay	0.0119	Glomerular filtration rate	0.0100
9	Sodium	0.0109	Discharge day	0.0096
10	Coefficient of variation of RDW	0.0108	Valsartan tablet	0.0086

LVEDD: left ventricular end diastolic diameter; RDW red blood cell distribution width.

## 4 Discussion

In this study, we explored the performance of four alternative methods based on conventional Cox regression or machine learning algorithms for survival analysis on the inpatient data from a secondary care institution in China, aiming to construct a framework of developing survival prediction models for risk of the readmission or mortality among hospitalized heart failure patients. Both discrimination and calibration were taken into account in the assessment of established models. Compared to the previous studies reporting the outcome of readmission or mortality of heart failure population as the end point [[3], [4], [5], [6], [7], [8], [9], [10], [11]], our study focused on both the overall indicators (c-index and integrated Brier score) and the temporal diversification of indicators (time-dependent AUC and time-dependent Brier score). The introduction of non-linear tree based survival models in our analysis broke through the limitation of constant proportional hazards in traditional generalized linear Cox regression, thus improving the clinical flexibility of models on the basis of time-variant hazard ratio assumption. These novel perspectives of our survival prediction modeling strategy ameliorated the knowledge deficiency in the field of current prognosis prediction among discharged heart failure patients.

Based on permutation importance of variables in different models, the factors’ contribution to readmission and mortality prediction among heart failure patients can be assessed. As traditional predictors for heart failure prognosis prediction, LVEDD, creatinine, systolic blood pressure, red blood cell count and uric acid also exhibited high predictive value in our machine learning models, which was consistent with the results for previous studies [[3], [4], [5], [6], [7], [8], [9], [10], [11]]. This indicates the crucial role of echocardiography, renal function and anemia evaluation in the follow-up management among heart failure population. Some prediction factors presenting important contribution for outcomes prediction in our machine learning models, such as mean platelet volume, coefficient of variation and standard deviation of red blood cell distribution width (RDW), were not reported in previous studies and not obviously related to prognosis based on clinical experience and medical knowledge. These factors might become potential predictors from the perspective of data mining and need to be confirmed through rigorous observational studies and clinical trials.

In the present study, we chose concordance index rather than integrated Brier score as the optimization target in model derivation. This strategy aimed to achieve higher concordance index in validation cohort via finding out a suitable hyperparameter combination. Since concordance index or AUC focuses on the categorical discrimination ability of a series of probability thresholds, while Brier score measures the deviation of observation value from the probabilities of positive category [30], inconsistency of model performance assessment between these two metrics might occur if there exists misclassification of high-confidence predictions and suffers severe penalty in Brier score calculation, just as the case in our traditional multivariate model in this study. As evaluation indicators, the concordance index and time-dependent AUC are more suitable for most clinical cases requiring category labels prediction such as individual risk stratification and clinical decision making. Nevertheless, in certain cases such as cost benefit analysis and public health policy formulation that the probability of overall prognosis of heart failure population is concerned for expectation calculation, Brier score might be preferred as assessment metrics and optimization target of machine learning models.

In derivation cohort, the non-linear tree-based ensemble algorithms, namely random survival forest and gradient boosting model, exhibited better discrimination than conventional linear model. However, when compared with c-index in validation cohort, the non-linear method didn't display superiority to linear model, indicating that the existence of over-fitting in tree-based ensemble algorithms might impair the stability and generalization of models in different populations [31]. Despite of failing to exceed linear model in prediction performance, non-linear models still showed values as they require smaller amount of prediction variables to realize equivalent prediction efficiency and give insight into factors' risk varying from time.

## 5 Limitations

Our analysis has several limitations. First, the study population was from a single clinic center and only internal validation was conducted in the present analysis. More external validation efforts are needed to confirm the models’ generalization ability in different hospital settings and in different districts with imbalanced health management levels. Auto machine learning based on Bayesian optimization seems to be a worthwhile approach for the generalization of the models to heterogeneous data source. Second, as a result of retrospective design of the study, there was a high proportion of missing values in some important predictors recognized in previous prospective studies. The lack of many echocardiographic variables, especially left ventricular ejection fraction (LVEF) considered as a highly relevant factor in prognosis evaluation in heart failure patient, occurred in almost half of the participants, resulting in severe information loss in model performance. The simple imputation strategy we adopted for filling missing value may inevitably introduce bias and other imputation methods, such as missForest [32], k-Nearest Neighbor and even multiple imputation [33,34], are worth trying in further analysis exploring a higher-quality model. Third, labeled as black-box model, the poor clinical interpretability of non-linear algorithms might lead to more ethical problems in clinical applications despite their better performance in particular settings.

## 6 Conclusion

In conclusion, we developed and validated machine learning survival prediction models among discharged heart failure patients in a Chinese cohort. The penalized Cox regression and non-linear machine learning survival analysis based on time-variant hazard ratio assumption had advantages over conventional Cox regression on the short-term probability prediction ability in model calibration. Non-linear models require less prediction variables to achieve equivalent discrimination and calibration ability compared with linear models if a meticulously designed feature selection strategy is adopted. While our study explored the application of non-linear algorithms integrated with survival analysis and built a framework for heart failure prognosis predicting using a particular structured tabular dataset, further research on fully utilizing of multimodal data such as medical documents with nature language and medical imaging for survival analysis via the popular technology of neural network and deep learning seems to be a promising direction to improve the model performance [35]. Moreover, introduction of automated machine learning methods is an extremely valuable issue in further researches to simplify modeling process and promote model application in heart failure prognosis prediction from different data sources [36].

## Author contribution

Rui Tong: Conceived and designed the experiments; Performed the experiments; Analyzed and interpreted the data; Contributed reagents, materials, analysis tools or data; Wrote the paper.

Zhongsheng Zhu: Conceived and designed the experiments; Analyzed and interpreted the data; Wrote the paper.

Jia Ling: Analyzed and interpreted the data; Contributed reagents, materials, analysis tools or data; Wrote the paper.

## Data availability statement

Data associated with this study has been deposited at https://doi.org/10.13026/8a9e-w734.

## Additional information

Supplementary content related to this article has been published online at [URL].

## Declaration of competing interest

The authors declare that they have no known competing financial interests or personal relationships that could have appeared to influence the work reported in this paper.
