# Supplementary material for: Comparison of linear and non-linear machine learning models for time-dependent readmission or mortality prediction among hospitalized heart failure patients
Source: Heliyon. 2023 May 6;9(5):e16068. doi: 10.1016/j.heliyon.2023.e16068 (PMC10192765; doi:10.1016/j.heliyon.2023.e16068)
Supplement: Supplementary Table 1 [file mmc1.docx]

The missing conditions of variables and the imputation method

| Variables | Missing number | Mission percentage(%) | Imputation method |
| --- | --- | --- | --- |
| destination.Home | 0 | 0 | Not needed |
| destination.Healthcare | 0 | 0 | Not needed |
| admission.ward.Cardiology | 0 | 0 | Not needed |
| admission.ward.GeneralWard | 0 | 0 | Not needed |
| admission.ward.ICU | 0 | 0 | Not needed |
| admission.way | 0 | 0 | Not needed |
| occupation.UrbanResident | 0 | 0 | Not needed |
| occupation.farmer | 0 | 0 | Not needed |
| occupation.officer | 0 | 0 | Not needed |
| occupation.worker | 0 | 0 | Not needed |
| discharge.department.Cardiology | 0 | 0 | Not needed |
| discharge.department.GeneralWard | 0 | 0 | Not needed |
| discharge.department.ICU | 0 | 0 | Not needed |
| visit.times | 0 | 0 | Not needed |
| gender | 0 | 0 | Not needed |
| body.temperature | 0 | 0 | Not needed |
| pulse | 0 | 0 | Not needed |
| respiration | 0 | 0 | Not needed |
| systolic.blood.pressure | 0 | 0 | Not needed |
| diastolic.blood.pressure | 0 | 0 | Not needed |
| weight | 4 | 0.2 | Imputed with mean |
| height | 4 | 0.2 | Imputed with mean |
| left.heart.failure | 0 | 0 | Not needed |
| right.heart.failure | 0 | 0 | Not needed |
| NYHA.cardiac.function.classification | 0 | 0 | Not needed |
| Killip.grade | 0 | 0 | Not needed |
| myocardial.infarction | 0 | 0 | Not needed |
| congestive.heart.failure | 0 | 0 | Not needed |
| peripheral.vascular.disease | 0 | 0 | Not needed |
| cerebrovascular.disease | 0 | 0 | Not needed |
| dementia | 0 | 0 | Not needed |
| chronic.obstructive.pulmonary.disease | 0 | 0 | Not needed |
| connective.tissue.disease | 0 | 0 | Not needed |
| peptic.ulcer.disease | 2 | 0.1 | Imputed with mode |
| diabetes | 0 | 0 | Not needed |
| moderate.to.severe.CKD | 2 | 0.1 | Imputed with mode |
| hemiplegia | 0 | 0 | Not needed |
| malignant.lymphoma | 0 | 0 | Not needed |
| solid.tumor | 0 | 0 | Not needed |
| liver.disease | 1 | 0.05 | Imputed with mode |
| AIDS | 0 | 0 | Not needed |
| type.II.respiratory.failure | 0 | 0 | Not needed |
| eye.opening | 0 | 0 | Not needed |
| verbal.response | 0 | 0 | Not needed |
| movement | 0 | 0 | Not needed |
| IMV | 0 | 0 | Not needed |
| NIMV | 0 | 0 | Not needed |
| FiO2 | 0 | 0 | Not needed |
| acute.renal.failure | 0 | 0 | Not needed |
| LVEF | 1344 | 68.0 | Imputed with median |
| LVEDD | 687 | 34.8 | Imputed with median |
| mitral.valve.EMS | 1018 | 51.5 | Imputed with median |
| mitral.valve.AMS | 1443 | 73.0 | Excluded |
| tricuspid.valve.return.velocity | 1201 | 60.8 | Imputed with median |
| tricuspid.valve.return.pressure | 1807 | 91.4 | Excluded |
| creatinine.enzymatic.method | 21 | 1.1 | Imputed with median |
| urea | 21 | 1.1 | Imputed with median |
| uric.acid | 21 | 1.1 | Imputed with median |
| glomerular.filtration.rate | 61 | 3.1 | Imputed with median |
| cystatin | 39 | 2.0 | Imputed with median |
| white.blood.cell | 25 | 1.3 | Imputed with median |
| monocyte.ratio | 25 | 1.3 | Imputed with median |
| red.blood.cell | 25 | 1.3 | Imputed with median |
| coefficient.of.variation.of.RDW | 27 | 1.4 | Imputed with median |
| standard.deviation.of.RDW | 27 | 1.4 | Imputed with median |
| mean.corpuscular.volume | 26 | 1.3 | Imputed with median |
| hematocrit | 26 | 1.3 | Imputed with median |
| lymphocyte.count | 25 | 1.3 | Imputed with median |
| mean.hemoglobin.volume | 26 | 1.3 | Imputed with median |
| mean.hemoglobin.concentration | 25 | 1.3 | Imputed with median |
| mean.platelet.volume | 101 | 5.1 | Imputed with median |
| basophil.ratio | 25 | 1.3 | Imputed with median |
| eosinophil.ratio | 25 | 1.3 | Imputed with median |
| hemoglobin | 26 | 1.3 | Imputed with median |
| platelet | 25 | 1.3 | Imputed with median |
| platelet.distribution.width | 101 | 5.1 | Imputed with median |
| platelet.hematocrit | 101 | 5.1 | Imputed with median |
| neutrophil.ratio | 25 | 1.3 | Imputed with median |
| D.dimer | 163 | 8.2 | Imputed with median |
| international.normalized.ratio | 32 | 1.6 | Imputed with median |
| activated.partial.thromboplastin.time | 31 | 1.6 | Imputed with median |
| thrombin.time | 31 | 1.6 | Imputed with median |
| prothrombin.activity | 43 | 2.2 | Imputed with median |
| prothrombin.time.ratio | 32 | 1.6 | Imputed with median |
| fibrinogen | 31 | 1.6 | Imputed with median |
| high.sensitivity.troponin | 75 | 3.8 | Imputed with median |
| myoglobin | 1580 | 80.0 | Excluded |
| carbon.dioxide.binding.capacity | 9 | 0.5 | Imputed with median |
| calcium | 9 | 0.5 | Imputed with median |
| potassium | 10 | 0.5 | Imputed with median |
| chloride | 9 | 0.5 | Imputed with median |
| sodium | 9 | 0.5 | Imputed with median |
| Inorganic.Phosphorus | 1574 | 79.7 | Excluded |
| serum.magnesium | 1574 | 79.7 | Excluded |
| hydroxybutyrate.dehydrogenase | 234 | 11.8 | Imputed with median |
| glutamic.oxaloacetic.transaminase | 246 | 12.4 | Imputed with median |
| creatine.kinase | 234 | 11.8 | Imputed with median |
| creatine.kinase.isoenzyme | 234 | 11.8 | Imputed with median |
| lactate.dehydrogenase | 234 | 11.8 | Imputed with median |
| brain.natriuretic.peptide | 33 | 1.7 | Imputed with median |
| high.sensitivity.protein | 1049 | 53.1 | Imputed with median |
| nucleotidase | 518 | 26.2 | Imputed with median |
| fucosidase | 518 | 26.2 | Imputed with median |
| albumin | 97 | 4.9 | Imputed with median |
| glutamyltranspeptidase | 97 | 4.9 | Imputed with median |
| glutamic.pyruvic.transaminase | 97 | 4.9 | Imputed with median |
| indirect.bilirubin | 97 | 4.9 | Imputed with median |
| alkaline.phosphatase | 97 | 4.9 | Imputed with median |
| globulin | 97 | 4.9 | Imputed with median |
| direct.bilirubin | 97 | 4.9 | Imputed with median |
| total.bile.acid | 499 | 25.3 | Imputed with median |
| erythrocyte.sedimentation.rate | 1673 | 84.7 | Excluded |
| cholesterol | 192 | 9.7 | Imputed with median |
| low.density.lipoprotein.cholesterol | 192 | 9.7 | Imputed with median |
| triglyceride | 192 | 9.7 | Imputed with median |
| high.density.lipoprotein.cholesterol | 192 | 9.7 | Imputed with median |
| homocysteine | 1834 | 92.8 | Excluded |
| apolipoprotein.A | 1804 | 91.3 | Excluded |
| apolipoprotein.B | 1804 | 91.3 | Excluded |
| lipoprotein | 1804 | 91.3 | Excluded |
| pH | 1000 | 50.6 | Imputed with median |
| standard.residual.base | 1000 | 50.6 | Imputed with median |
| standard.bicarbonate | 1000 | 50.6 | Imputed with median |
| partial.pressure.of.carbon.dioxide | 1000 | 50.6 | Imputed with median |
| total.carbon.dioxide | 1000 | 50.6 | Imputed with median |
| methemoglobin | 1001 | 50.7 | Imputed with median |
| reduced.hemoglobin | 1001 | 50.7 | Imputed with median |
| glucose.blood.gas | 1001 | 50.7 | Imputed with median |
| lactate | 1000 | 50.6 | Imputed with median |
| measured.residual.base | 1000 | 50.6 | Imputed with median |
| measured.bicarbonate | 1000 | 50.6 | Imputed with median |
| carboxyhemoglobin | 1001 | 50.7 | Imputed with median |
| oxygen.saturation | 1005 | 50.9 | Imputed with median |
| partial.oxygen.pressure | 1005 | 50.9 | Imputed with median |
| oxyhemoglobin | 1001 | 50.7 | Imputed with median |
| anion.gap | 1000 | 50.6 | Imputed with median |
| dischargeDay | 0 | 0 | Not needed |
| ageCat | 0 | 0 | Not needed |
| Furosemide tablet | 0 | 0 | Not needed |
| Spironolactone tablet | 0 | 0 | Not needed |
| Milrinone injection | 0 | 0 | Not needed |
| Furosemide injection | 0 | 0 | Not needed |
| Deslanoside injection | 0 | 0 | Not needed |
| Digoxin tablet | 0 | 0 | Not needed |
| Torasemide tablet | 0 | 0 | Not needed |
| Valsartan Dispersible tablet | 0 | 0 | Not needed |
| Isosorbide Mononitrate tablet | 0 | 0 | Not needed |
| Atorvastatin calcium tablet | 0 | 0 | Not needed |
| Shenfu injection | 0 | 0 | Not needed |
| Benazepril hydrochloride tablet | 0 | 0 | Not needed |
| Hydrochlorothiazide tablet | 0 | 0 | Not needed |
| sulfotanshinone sodium injection | 0 | 0 | Not needed |
| Nitroglycerin injection | 0 | 0 | Not needed |
| Isoprenaline Hydrochloride injection | 0 | 0 | Not needed |
| Dobutamine hydrochloride injection | 0 | 0 | Not needed |
| Adenosine Cyclophosphate injection | 0 | 0 | Not needed |
