# Supplementary material for: Comparison of linear and non-linear machine learning models for time-dependent readmission or mortality prediction among hospitalized heart failure patients
Source: Heliyon. 2023 May 6;9(5):e16068. doi: 10.1016/j.heliyon.2023.e16068 (PMC10192765; doi:10.1016/j.heliyon.2023.e16068)
Supplement: Supplementary Table 2 [file mmc2.docx]

Cox regression analysis of the risk of readmission or mortality among heart failure patients

| Variables | Univariate analysis | | Multivariate analysis | |
| --- | --- | --- | --- | --- |
|  | HR(95% CI) | P | Adjusted HR(95% CI) | P |
| Occupation: Farmer | 0.581(0.403-0.837) | 0.004 | 0.877(0.630-1.222) | 0.439 |
| Visit times | 1.382(1.110-1.719) | 0.004 | 1.091(0.828-1.438) | 0.536 |
| Pulse | 0.994(0.990-0.999) | 0.018 | 0.999(0.994-1.004) | 0.609 |
| Systolic blood pressure | 0.992(0.988-0.996) | <0.001 | 0.998(0.992-1.003) | 0.398 |
| Diastolic blood pressure | 0.987(0.980-0.994) | <0.001 | 1.000(0.990-1.009) | 0.948 |
| Right heart failure | 1.526(1.196-1.947) | <0.001 | 1.109(0.881-1.397) | 0.378 |
| NYHA classification | 1.302(1.132-1.497) | <0.001 | 1.050(0.897-1.229) | 0.542 |
| Killip grade | 1.159(1.017-1.322) | 0.027 | 1.030(0.889-1.192) | 0.695 |
| Congestive heart failure | 0.635(0.453-0.889) | 0.008 | 0.820(0.542-1.241) | 0.348 |
| Diabetes | 1.386(1.127-1.705) | 0.002 | 1.053(0.829-1.339) | 0.671 |
| Moderate to severe CKD | 1.489(1.212-1.830) | <0.001 | 1.074(0.818-1.441) | 0.607 |
| Eye opening | 0.696(0.503-0.964) | 0.029 | 1.133(0.458-2.801) | 0.787 |
| Verbal response | 0.786(0.639-0.967) | 0.023 | 0.893(0.517-1.541) | 0.683 |
| IMV | 2.317(1.271-4.221) | 0.006 | 1.320(0.308-5.659) | 0.709 |
| Acute renal failure | 4.753(1.773-12.742) | 0.002 | 1.962(0.428-8.999) | 0.386 |
| LVEDE | 1.013(1.002-1.023) | 0.016 | 1.004(0.992-1.016) | 0.487 |
| Creatinine | 1.002(1.001-1.003) | <0.001 | 1.001(0.999-1.003) | 0.436 |
| Urea | 1.036(1.020-1.051) | <0.001 | 0.995(0.966-1.024) | 0.714 |
| Uric acid | 1.001(1.001-1.002) | <0.001 | 1.000(0.999-1.001) | 0.547 |
| GFR | 0.993(0.990-0.996) | <0.001 | 1.001(0.996-1.005) | 0.757 |
| cystatin | 1.215(1.108-1.333) | <0.001 | 0.927(0.759-1.131) | 0.455 |
| Red blood cell | 0.758(0.672-0.854) | <0.001 | 0.964(0.500-1.860) | 0.914 |
| Coefficient of variation of RDW | 1.072(1.027-1.120) | 0.002 | 1.009(0.854-1.192) | 0.918 |
| Standard deviation of RDW | 1.029(1.016-1.043) | <0.001 | 1.007(0.959-1.058) | 0.767 |
| Hematocrit | 0.072(0.019-0.271) | <0.001 | 1.138(7.0*10^-6^-1.9*10^5^) | 0.983 |
| Mean platelet volume | 0.926(0.877-0.978) | 0.006 | 0.986(0.928-1.047) | 0.642 |
| Hemoglobin | 0.993(0.990-0.997) | <0.001 | 1.000(0.970-1.030) | 0.976 |
| Carbon dioxide binding capacity | 0.975(0.956-0.995) | 0.013 | 1.008(0.979-1.039) | 0.591 |
| Potassium | 1.284(1.131-1.459) | <0.001 | 1.032(0.868-1.228) | 0.718 |
| Sodium | 0.974(0.956-0.992) | 0.006 | 0.997(0.973-1.021) | 0.780 |
| Glutamic oxaloacetic transaminase | 1.000(1.000-1.001) | 0.007 | 1.000(0.996-1.001) | 0.623 |
| cholesterol | 0.908(0.828-0.997) | 0.043 | 1.012(0.828-1.237) | 0.906 |
| LDL-C | 0.865(0.756-0.990) | 0.035 | 0.986(0.739-1.316) | 0.924 |
| pH | 0.089(0.014-0.542) | 0.009 | 7.3*10^-5^ (8.3*10^-17^-6.4*10^7^) | 0.497 |
| Standard residual base | 0.956(0.930-0.982) | 0.001 | 1.944(0.324-11.657) | 0.466 |
| Standard bicarbonate | 0.939(0.904-0.975) | 0.001 | 1.250(0.712-2.192) | 0.437 |
| Total carbon dioxide | 0.958(0.931-0.986) | 0.004 | 0.997(0.152-6.559) | 0.997 |
| Lactate | 1.096(1.027-1.169) | 0.006 | 0.982(0.868-1.111) | 0.776 |
| Measured residual base | 0.947(0.919-0.976) | <0.001 | 0.770(0.319-1.855) | 0.560 |
| Measured bicarbonate | 0.955(0.927-0.985) | 0.003 | 0.541(0.030-9.810) | 0.678 |
| Carboxyhemoglobin | 0.728(0.563-0.941) | 0.015 | 0.915(0.713-1.173) | 0.481 |
| Discharge day | 1.019(1.006-1.031) | 0.003 | 1.003(0.988-1.020) | 0.667 |
| Age | 1.146(1.051-1.249) | 0.002 | 1.039(0.937-1.152) | 0.468 |
| Milrinone injection | 1.316(1.088-1.593) | 0.005 | 0.987(0.786-1.240) | 0.912 |
| Furosemide injection | 1.447(1.080-1.937) | 0.013 | 1.045(0.772-1.413) | 0.777 |
| Valsartan Dispersible tablet | 1.269(1.011-1.593) | 0.040 | 1.114(0.865-1.434) | 0.405 |
| Shenfu injection | 1.330(1.052-1.681) | 0.017 | 1.005(0.765-1.320) | 0.972 |
| Hydrochlorothiazide tablet | 0.696(0.524-0.925) | 0.013 | 0.981(0.739-1.300) | 0.892 |
